# Platelet monoamine oxidase activity predicts alcohol sensitivity and voluntary alcohol intake in rhesus monkeys

**DOI:** 10.3109/03009731003605813

**Published:** 2010-03-10

**Authors:** Hanna-Linn Wargelius, Claudia Fahlke, Stephen J. Suomi, Lars Oreland, James Dee Higley

**Affiliations:** ^1^Department of Neuroscience, Uppsala University, UppsalaSweden; ^2^Department of Psychology, Göteborg University, GöteborgSweden; ^3^Laboratories of Comparative Ethology and Clinical Studies—Primate Unit, National Institutes of Health Animal Center, Poolesville, MarylandUSA; ^4^Department of Psychology, Brigham Young University, Provo, UtahUSA

**Keywords:** Aggressiveness, alcohol sensitivity, MAO-B, platelet, rhesus macaque

## Abstract

Platelet monoamine oxidase B (MAO-B) has been proposed to be a biological marker for the properties of monoamine systems, with low activity being associated with vulnerability for high scores on personality traits such as sensation seeking, monotony avoidance, and impulsiveness, as well as for vulnerability for alcoholism. In the present study, platelet MAO-B activity was analysed in 78 rhesus macaques, and its relation to voluntary alcohol intake and behaviours after intravenous alcohol administration was observed.

Monkeys with low platelet MAO-B activity had low levels of 5-hydroxyindole acetic acid in cerebrospinal fluid and showed excessive aggression after alcohol administration. A novel finding was that animals with low platelet MAO-B activity showed less intoxication following alcohol administration. As we have shown previously, they also voluntarily consumed more alcohol. We here replicate results from studies on both humans and non-human primates, showing the utility of platelet MAO as a marker for risk behaviours and alcohol abuse. Furthermore, we link platelet MAO activity to alcohol sensitivity.

## Introduction

Monoamine oxidases (MAO-A and MAO-B) are enzymes that oxidatively deaminate both endogenous and exogenous monoamines. MAO-A primarily acts on noradrenaline and serotonin, while MAO-B prefers the trace amine phenylethylamine. Dopamine is equally preferred by both enzymes. Both MAO forms are abundant in the primate brain; however, platelet MAO is solely of the B-type (1). The amino acid sequence of MAO-B cDNA in different tissues is identical (2). However, a correlation between platelet and brain MAO-B activity has not been established, hence platelet MAO activity should probably not be regarded as a peripheral marker of general MAO-B activity in the brain.

In 1975 Gottfries et al. reported low MAO-B activity in post-mortem brains of alcoholics who had committed suicide (3). Shortly afterwards, it was found that low platelet MAO activity was associated with aspects of personality traits often seen in some types of alcoholics (4). Similarly, it was shown that alcoholics had low levels of MAO activity in platelets (5,6). Since then, low platelet MAO activity has, as a result of several studies, been proposed to be a biological marker for vulnerability for alcoholism, in particular for ‘type II alcoholism’ which is characterized by a heavy genetic load, drinking at an early age, impaired impulse control, antisocial personality traits, and aggressive behaviour (7). There seems to be no effect on platelet MAO itself from long-term alcohol intake since chronic exposure to alcohol does not alter MAO activity in rats (8). Low platelet MAO activity has also been linked to low central nervous system (CNS) monoamine turn-over, as measured by low monoamine metabolite levels in cerebrospinal fluid (CSF) (9–11). Variations in activity of central monoamine systems stand out as frequently repeated findings in several studies on alcoholism and as being an important factor for the expression of certain behavioural traits (12). This is in line with our long-standing hypothesis that platelet MAO-B activity is a marker for the constitutional properties of central monoamine systems (see (13)).

Low level of response to alcohol has been pointed out as a predictor for future heavy drinking in humans (14,15). There is also evidence from animal studies suggesting that a less pronounced response to alcohol is associated with higher alcohol consumption and that alcohol sensitivity to some degree is heritable (16,17). Development of alcohol tolerance, which is an important process in the development of alcoholism, has in rodents been associated with initially less sensitivity to alcohol (18). Furthermore, alcoholics often report that, compared to their friends, when they started drinking, large amounts of alcohol were necessary to get desired effects (19). It has been demonstrated that low CSF 5-hydroxyindole acetic acid (5-HIAA) is associated with lower sensitivity to alcohol in rhesus monkeys, and there are also other studies linking a weak serotonin system to high tolerance to the effects of alcohol (20–22). To our knowledge, there are no studies on whether subjects who are less sensitive to the effects of alcohol are also low in platelet MAO-B activity.

A problem in association studies with regard to platelet MAO-B has been that compounds in cigarette smoke have an inhibitory effect on MAO activity (23,24), and since alcoholics very often are smokers (25–27) it has been argued that the lower platelet MAO activity seen in alcohol-dependent individuals could be an effect of smoking (28,29). However, yet other studies do find associations even when controlling for smoking (13,30), which has been confirmed in rhesus monkeys (11). In order to avoid confounding factors, such as smoking or previous alcohol use, we used a model of free-living alcohol-naive rhesus macaques to explore relations between platelet MAO-B activity, sensitivity to the behavioural effects of alcohol, as well as voluntary alcohol intake.

## Material and methods

### Subjects

A total of 78 rhesus macaques (*Macaca mulatta*) (31 males and 47 females) group-housed at the National Institutes of Health Animal Center in Poolesville, MD, USA were used in this study. The monkeys were young alcohol-naive adolescents and members of an on-going longitudinal study (22). The cohort used in the previous study by Fahlke et al. (11) was not included in the present study.

The research protocol was approved by the National Institutes of Health, Bethesda, MD, USA in accordance with and as required by the Animal Welfare Act.

### Alcohol administration procedure and ethanol sensitivity

#### Intravenous (IV) alcohol administration

All subjects of the same gender were given identical IV dosages of a 16.8% ethanol solution (for females 2.0 g, for males 2.2 g ethanol per kg body-weight) into the saphenous vein. These doses produce a blood alcohol concentration of 250 mg/100 mL, a level that is about three times higher than the legal level of intoxication for most of the USA.

#### Behavioural assessment

After the intravenous ethanol administration, trained personnel scored the monkey's degree of intoxication. Three observers subjectively rated the animals' general degree of intoxication. Firstly the subject's general motor behaviour was scored (fall, bump against wall, sway), and thereafter provoked aggressive behaviour in response to a human investigator was assessed for 5 minutes (open mouth threats, stares) (see Table I). The duration (in seconds) of stare threat and open mouth threat were recorded, while the remaining behaviours were recorded as frequencies. For detailed protocols see Schwandt et al. (31).

**Table I.** Description of behaviours registered after intravenous ethanol administration.

Behaviour	Definition
Fall	Animal loses balance and involuntarily drops to the floor
Hit wall	The animal's body hits the wall of the room
Sway	The body veers in any direction out of control
Stare threat	Intense staring with eyes wide open
Open mouth threat	Tense opening of the mouth forming an O
Aggression	Sum of: duration of Stare threat and Open mouth threat

### Alcohol consumption

#### Alcohol self-administration

The animals' voluntary alcohol intake was assessed using an oral ethanol self-administration procedure where the animals had free access to an aspartame-sweetened 8.4% ethanol solution, an aspartame vehicle, and home-cage water, 5 days a week during the 2-week experimental phase. The animals were kept in their home-cage environment. The home-cage water was turned off for 1 hour prior to alcohol exposure, to preclude water satiation. Thereafter, during the experimental period, water was freely available. A more detailed protocol is described in Higley et al. (32).

### CSF sample collection and enzyme activity measurement

CSF was obtained directly via cisternal puncture on anaesthetized animals. Gas chromatography mass spectrometry was used to analyse CSF for concentrations of neurotransmitter metabolites (5-HIAA and homovanillic acid (HVA)) (22).

Platelet MAO-B activity was measured using a modified protocol (33). In short, blood samples of approximately 4 mL were drawn into Vacutainer^®^ tubes containing ethylenediamine tetra-acetic acid (EDTA). From the blood sample, platelet-rich plasma was prepared by low-speed centrifugation. Thereafter, platelet concentrations of the plasma samples were estimated electronically, and the plasma was stored at −80°C. Catalytic activity of platelet MAO-B was analysed by a radiometric assay with ^14^C-labelled 2-phenylethylamine (β-PEA) as substrate. Enzyme activity is expressed as nmol of substrate oxidized per 10^10^ platelets per minute. All samples were analysed blindly and in duplicate. Blood samples and CSF samples were taken before the alcohol administration.

### Statistics

For statistical analyses, the animals were divided into a high MAO-B activity group and a low MAO-B activity group, using median split.

Linear regression analysis was used to compare MAO activity and CSF metabolite concentrations. One-way ANOVA and *t* test were applied to compare alcohol-induced behaviours and alcohol consumption between the two MAO activity groups.

The statistical analyses were performed with the SPSS 11 software.

Protocols for the care and use of experimental animals were approved by the Institutional Animal Care and Use Committee of the National Institute on Alcohol Abuse and Alcoholism and the National Institute of Child Health and Human Development, National Institutes of Health.

## Results

Platelet MAO activity ranged from 0.53 to 1.81 nmol of substrate oxidized per 10^10^ platelets per minute. There was no difference in platelet MAO activity between the sexes, thus males and females were analysed together (mean nmol of substrate oxidized per 10^10^ platelets per minute was in males 1.20, SEM 0.049, and in females 1.19, SEM 0.038).

**Figure 1.**
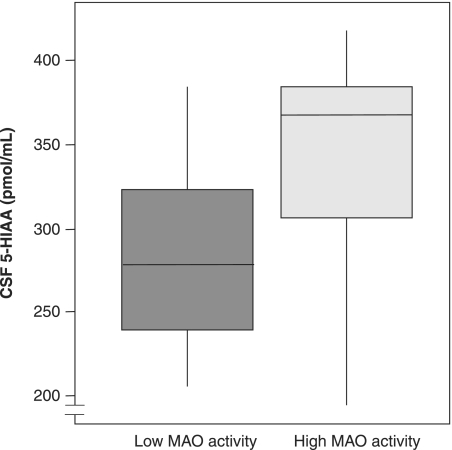
CSF 5-HIAA levels were lower in animals with low platelet MAO-B activity (*t* = −2.39, *P* <0.05). Box plot with whiskers showing largest and smallest observation. The boxes represent the lower and the upper quartiles with the median value marked as a line within the boxes.

**Figure 2.**
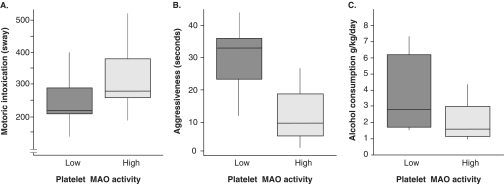
A: Motoric intoxication scores were lower in monkeys with low platelet MAO-B activity than in monkeys with high platelet MAO-B activity (*t* = −2.10, *P* <0.05). Reported as frequency. B: Monkeys with low platelet MAO-B activity showed more aggressive behaviour after alcohol infusion (*t* = 4.55, *P* <0.001). Stare threats. C: Monkeys with low platelet MAO-B activity consumed more alcohol than monkeys with high MAO-B activity (*t* = 2.15, *P* <0.05). Mean alcohol intake per day during the two-weeks period. Box plots with whiskers showing largest and smallest observation. The boxes represent the lower and the upper quartiles with the median value marked as a line within the boxes.

Animals with low platelet MAO activity were low in CSF 5-HIAA (*t* = −2.39, *P* <0.05) (Figure 1) and showed less intoxication following alcohol administration than did animals with high platelet MAO activity (*t* = −2.10, *P* <0.05). Hence, they scored lower frequencies on the parameters hit wall, sway, and fall (Figure 2A). The animals in the low MAO activity group showed more ethanol-induced aggression (*t* = 4.55, *P* <0.001) in terms of longer duration of stare threat and open mouth threat (Figure 2B). Total frequency of aggressiveness scores was also higher in animals with low platelet MAO activity (*t* = 2.42, *P* <0.02). These monkeys also drank more alcohol in the voluntary alcohol intake paradigm (*F* = 4.62, *P* <0.05) (Figure 2C). There was a positive correlation between levels of the metabolites 5-HIAA and HVA (*r* = 0.80, *P* <0.001).

## Discussion

The results in the present study support previous findings of platelet MAO-B activity being a biological marker for behaviours that are linked to alcohol abuse. Monkeys with low platelet MAO activity were low in CSF 5-HIAA and showed less intoxication following alcohol administration. Hence, low platelet MAO activity was associated with lower levels of ataxia. However, at the same time these animals seemed to be more disinhibited, which was expressed as an increase in alcohol-induced aggression. In addition, the animals with platelet MAO activity below the median had higher voluntary alcohol intake.

The inhibition of MAO by cigarette smoking has been put forward as a confounding factor in studies of associations between alcoholism and MAO activity (28). However, the present study replicates the findings of Fahlke and colleges who showed that (non-smoking) rhesus monkeys that exhibit type II-like alcohol features have low platelet MAO activity (11). Similarly to the results of the current study, they also found that platelet MAO activity correlated positively with CSF 5-HIAA concentrations. These results are in line with human studies on CSF 5-HIAA in alcoholics (34). Central monoamine system variations, as measured by serotonin metabolite concentration and serotonin transporter availability, have been associated with sensitivity to alcohol intoxication in both humans and monkeys (20–22,35). In a series of studies, Schuckit and co-workers found that low level of response to alcohol is associated with increased risk for alcohol use disorders (36). In what is, as far as we know, the first demonstration of an association between MAO and levels of intoxication, in the present study we link alcohol sensitivity to platelet MAO activity. Moreover, we found that 5-HIAA levels were positively correlated with levels of HVA. This suggests that platelet MAO activity not only reflects constitutional properties of the serotonergic system but also of the dopaminergic system. Similar results with regard to serotonergic and dopaminergic activities have been found in aggressive behaviour (37,38). Alcohol is generally seen as a causative factor in violent behaviour (39). It has been noted that alcohol potentiates aggressive behaviour in some (40–42) but not in all individuals (43,44). Thus, some individuals seem to have a greater sensitivity for the aggression-related (disinhibition-related) behavioural effects of alcohol. Serotonin is generally looked upon as an inhibitor of behaviours, which is supported by molecular genetic studies showing that, in animals, aggressive behaviour can be elicited by manipulation of the serotonergic system (45–48). Furthermore, low levels of CSF 5-HIAA have previously been linked to low brain serotonin turn-over and aggressive behaviour in monkeys (22).

The results regarding alcohol-mediated aggression and platelet MAO-B activity are also in line with the notion of a stronger evoked response in individuals with low platelet MAO-B (49). Low serotonergic tone is associated with higher event-related potentials in auditory and visual evoked potential-settings (50). This high neuronal responsiveness in individuals with low 5-HIAA could possibly relate to the high emotional responsiveness in forms of aggression in subjects with low platelet MAO activity in the present report.

Personality traits, such as impulsiveness and sensation seeking, and related behaviours are the results of gene–environment interactions, and platelet MAO-B activity is likely to act as an endophenotype in this regard. Both platelet MAO-B activity and CSF levels of 5-HIAA are to a large extent genetically controlled (51,52). The absence of obvious correlations between platelet MAO-B activity and general MAO-B activity in the adult brain (53,54) makes it tempting to speculate that platelet MAO-B activity reflects processes of importance for the development of e.g. the serotonergic system during foetal life. These processes could involve the activities of transcription factors for a variety of proteins, including MAO-B, constituting the monoamine systems (see (55)). Alternatively, foetal brain MAO activities might regulate brain serotonin levels, which, in turn, act as an important modulator of neuronal growth during foetal brain development (see (56)).

Together with other findings, our results support the hypothesis that individuals with low platelet MAO-B activity have central monoamine systems with lower turn-over, rendering them more vulnerable for behavioural and psychiatric disturbances, e.g. alcoholism (13,57). In human studies it is often difficult to tell whether certain alcohol-mediated behaviours occur as a result of differences in alcohol intake, if they are related to different pharmacological effects of alcohol, or whether they are provoked by situational factors. An advantage of the rhesus monkey model used is that all study subjects were naive to alcohol, whereas studies with human participants often suffer from problems in determining whether a low response to alcohol is a consequence of tolerance due to previous alcohol use, or if it is the result of a different initial sensitivity to alcohol.
